# Phenolic Composition and Antioxidant Properties of Bee Bread Collected in Three Consecutive Beekeeping Seasons in Poland

**DOI:** 10.3390/molecules31020304

**Published:** 2026-01-15

**Authors:** Teresa Szczęsna, Katarzyna Jaśkiewicz, Natalia Skubij, Jacek Jachuła

**Affiliations:** The National Institute of Horticultural Research, Konstytucji 3 Maja 1/3, 96-100 Skierniewice, Poland; katarzyna.jaskiewicz@inhort.pl (K.J.); natalia.skubij@inhort.pl (N.S.); jacek.jachula@inhort.pl (J.J.)

**Keywords:** bee products, phenolic compounds, phenolic acids, flavonoids, DPPH, TPC, HPLC-DAD

## Abstract

Bee bread contains numerous bioactive compounds, including phenolic compounds, which have been associated with antioxidant properties. In this study, we determined the phenolic composition of Polish bee bread collected over three consecutive years using HPLC-DAD. We also measured total phenolic content (TPC) and antioxidant activity, expressed as DPPH radical scavenging activity. The highest concentrations were observed for *p*-coumaric, *trans*-ferulic, and caffeic acids, as well as for two flavonoids—rutin and hesperidin. The contents of individual phenolic compounds varied across the years of sample collection, with the exception of *p*-coumaric and vanillic acids. Despite year-to-year differences in TPC, no significant correlation with antioxidant activity (>90% in all samples) was observed, indicating a substantial contribution of non-phenolic compounds to antioxidant capacity. Principal Component Analysis revealed that almost all samples clustered into three groups according to their year of collection. We conclude that the year-to-year variation in phenolic compound content in bee bread is likely attributable to differences in available pollen sources. Our findings expand the current knowledge of the nutritional value of bee bread produced in Poland and strengthen the premises for its use as a functional food.

## 1. Introduction

In recent years, special attention has been directed toward antioxidant compounds naturally present in foods. Polyphenolic compounds are considered major contributors to the antioxidant capacity of foods [1,2]. One of the most important properties of phenolic compounds—especially polyphenols—is their ability to scavenge reactive oxygen species (ROS) and reactive nitrogen species (RNS) that damage lipids, proteins, DNA, and RNA. The mechanism of free-radical inactivation by phenolic compounds typically involves hydrogen atom transfer (HAT) and single electron transfer (SET). Both mechanisms generate phenoxyl radicals, which can subsequently undergo rearrangement or react with each other to form dimers or higher-order polymers of the original phenolic compounds—structures that pose a lower risk to cells. Although polyphenols are widespread in the plant kingdom, humans and animals are unable to synthesize them and obtain them from diet [3,4].

Polyphenols are particularly important in the prevention of civilization diseases, such as cardiovascular disorders, cancer, diabetes, hypertension, and neurodegenerative conditions, which are considered to result from accumulation of reactive oxygen species and consequent oxidative stress [5]. Numerous studies have demonstrated that diets rich in polyphenols can help prevent the onset of several civilization diseases [2,4,6,7,8,9].

Bee products, including bee bread, are natural sources of polyphenolic compounds [10,11,12,13,14,15,16]. Bee bread is derived from floral pollen collected and processed by honey bees and subsequently stored in wax combs. To produce bee bread, bees mix pollen with nectar and enzymatic secretions from their digestive glands, and then deposit the mixture in the hive under anaerobic conditions and seal it with a layer of honey. During storage, various microorganisms—primarily lactic acid bacteria—drive a fermentation process that, together with enzymatic transformations, breaks down the original pollen and nectar components. As a result, bee bread becomes enriched with a wide range of nutrients, including carbohydrates, amino acids, unsaturated fatty acids, vitamins, micro- and macroelements, and diverse bioactive substances such as polyphenols [10,11,12,13,14,15,17,18,19,20,21].

Bee bread is widely recognized as a valuable natural source of antioxidants, with its high antioxidant potential primarily attributed to polyphenolic constituents [22,23,24,25,26,27,28]. In numerous studies, the antioxidant capacity of bee bread has been evaluated using a variety of analytical methods, including total phenolic content (TPC), total flavonoid content (TFC), 2,2-diphenyl-1-picrylhydrazyl (DPPH), 2,2-azinobis-(3-ethylbenzothiazoline-6-sulfonic acid) (ABTS), and ferric reducing antioxidant power (FRAP) assays. The antioxidant activity of bee bread varies widely across countries of origin. Reported values for DPPH+ radical scavenging range from 0.98 to 2.39 mg/mL (EC_50_), while ABTS scavenging activity ranges from 0.50 to 2.15 mg/mL (EC_50_). In addition, FRAP values have been reported between 0.02 and 0.43 mmol Trolox/g. The total phenolic content (TPC) of bee bread spans from 195 to 4340 mg GAE/100 g, whereas total flavonoid content (TFC) ranges from 25.6 to 350.4 mg quercetin/100 g [12,13,14,17,19,21,23,25,28].

A wide range of phenolic acids—including cinnamic, *p*-coumaric, ferulic, caffeic, chlorogenic, vanillic, syringic, gallic, ellagic, sinapic, abscisic, and salicylic acids—and numerous flavonoids, such as catechin, epicatechin, quercetin, apigenin, galangin, kaempferol, isorhamnetin, chrysin, pinocembrin, hesperetin, hesperidin, naringenin, luteolin, rutin, and tricetin, have been identified in bee bread by various authors [26,27,28,29,30,31,32,33].

Due to its beneficial effects on the human immune system, as well as its antibiotic and antioxidant properties and greater bioavailability, bee bread is increasingly sought by consumers [19,21,34]. However, production of bee bread in apiaries remains limited, primarily due to factors such as the high labor costs involved. Bee bread yield depends on the intensity of pollen supply and the specific colony management methods employed [35]. Preliminary studies suggest that laboratory-scale production may offer an alternative, using bacteria such as *Lactobacillus rhamnosus*, *Lactobacillus plantarum*, or yeasts *Saccharomyces cerevisiae* to ferment bee pollen [36,37]. These studies demonstrated that, compared to natural bee bread, fermented pollen can exhibit higher polyphenol content while maintaining comparable antioxidant activity.

Consumer awareness of so-called functional foods—i.e., foods that exert beneficial, pro-health effects on the human body, primarily due to their natural content of biologically active compounds—is steadily increasing. Given the nutritional value and biological potential of bee bread, numerous studies have aimed to characterize samples from different regions, with a focus on identifying distinctive features and samples exhibiting elevated levels of specific compounds [12,13,14,22,23].

To date, research on bee bread collected under Polish climatic conditions—particularly regarding its antioxidant activity and phenolic compound profile—has been limited. Only a few studies are available in the literature, and these have typically focused either on extensive analyses of single bee bread samples or on investigations involving a relatively small number of samples (up to 19) [25,28,29,38]. In the present work, we investigated both the antioxidant properties and the qualitative and quantitative composition of phenolic compounds in bee bread samples collected over three consecutive beekeeping seasons. Additionally, we analyzed the relationship between the content of specific phenolic compounds and the antioxidant capacity of bee bread. These data may serve as a valuable foundation for future research on the potential use of bee bread as a functional food.

## 2. Results and Discussion

### 2.1. Phenolic Compound Profile

Our study focused on the phenolic compounds most frequently and abundantly reported in bee bread samples and other bee products from different regions, as described by previous authors [12,13,14,30,31,32]. The analytical capabilities of our laboratory, based on HPLC-DAD, enabled the determination of 12 phenolic compounds in bee bread samples collected over three consecutive beekeeping seasons. Four phenolic acids (caffeic, *p*-coumaric, *trans*-ferulic, and vanillic), vanillin aldehyde, and six flavonoids (hesperidin, rutin, quercetin, kaempferol, hesperetin, and isorhamnetin) were identified and quantified in all samples. In 2015, salicylic acid was detected only in two samples. The quantitative results for the individual phenolic acids, vanillin aldehyde and flavonoids in each of the bee bread samples are presented in Supplementary Table S1, while mean values for each collection season are given in Table 1. It should be emphasized that the bee bread samples collected over the three consecutive beekeeping seasons originated from honey bee colonies overwintering each year in the same apiary. Each spring, the colonies were transported to various locations within a radius of approximately 100 km from the overwintering site to enhance bee bread production by increasing the diversity of pollen sources available to the bees. The primary pollen sources contributing to bee bread production were *Salix* spp., *Taraxacum officinale*, *Ribes nigrum*, *Prunus domestica*, *Malus domestica*, *Brassica napus* var. *oleifera*, and *Rubus idaeus* [35].

Numerous studies have examined the phenolic composition of bee bread. When comparing the phenolic profiles reported in this study with those presented by other authors, it is important to note that different chromatographic techniques have been employed across studies (HPLC-UV, HPLC-DAD, HPLC-PDA, HPLC-DAD-ESI/MS, UHPLC-MS/MS, UPLC-PDA-MS/MS, LC-MS/MS, GC-MS). These methodological differences influence both the qualitative and quantitative characterization of polyphenols in bee bread. Among these techniques, HPLC-DAD is the most widely used for the identification and quantification of individual phenolic compounds in bee bread [10,12,13,14,20,21,23,27,28,29,30,31,32].

Among the phenolic acids analyzed in our study, *p*-coumaric acid exhibited the highest mean concentration. Its levels were consistent across all beekeeping seasons, ranging from a mean value of 195.02 µg/g in 2015 to 274.63 µg/g in 2016. Our results for *p*-coumaric acid were substantially higher than those reported by several authors. Ilie et al. [14] found *p*-coumaric acid concentrations ranging from 58.02 to 109.13 µg/g in twelve bee bread samples collected from seven districts of Romania. According to Kolayli et al. [23], *p*-coumaric acid was among the dominant phenolic acids in bee bread from various regions of Turkey, with levels between 7.35 and 71.39 µg/g. Can et al. [13] also detected *p*-coumaric acid in all bee bread samples from different regions of Turkey, at concentrations from 10.511 to 95.407 µg/g. Aksoy et al. [12] did not detect *p*-coumaric acid in samples collected from five locations in the Ardahan province of Turkey, and only a trace amount (0.02 µg/g) was found in the sixth location. In contrast, Bayram et al. [33] reported considerably higher amounts of the acid, ranging from 2870 to 14,244 µg/g in five locations in Turkey. In an earlier study, Isidorov et al. [29] showed that bee bread from Poland, Ukraine and Russia contained substantial amounts of *p*-coumaric acid (1000–4000 µg/g).

Our results show that the average *trans*-ferulic acid content depended on the collection season of the bee bread samples. The highest mean level was recorded in samples collected in 2016 (187.15 µg/g), while the lowest was found in those from 2017 (40.73 µg/g). Studies by Aksoy et al. [12] reported much lower ferulic acid concentrations in bee bread, ranging from 0.25 to 3.25 µg/g. In Romanian samples, Ilie et al. [14] found ferulic acid levels between 7.00 and 41.46 µg/g. Research on bee bread from different regions of Turkey showed that ferulic acid was detected in only one sample at 13.77 µg/g [23]. Similarly, Can et al. [13] reported that among fifteen samples from other Turkish regions, only one contained ferulic acid, at 10.985 µg/g. Isidorov et al. [29] detected only trace amounts of ferulic acid in bee bread from several countries, including Poland. Bayram et al. [33] observed high variability in *trans*-ferulic acid content across Turkish samples, with values ranging from 2.47 µg/100 g to 96.10 µg/100 g. In contrast, Elsayed et al. [27] reported high amounts of ferulic acid (207.40 µg/g) in bee bread collected in Egypt during the citrus blooming period.

We found the highest mean caffeic acid content in bee bread samples collected during the 2016 season (111.85 µg/g). Samples from 2015 and 2017 contained, on average, approximately 40% lower amounts of this acid. Our results are comparable with those reported by Ilie et al. [14], who found that caffeic acid was one of the major phenolic acids in bee bread and its concentration in samples of different palynological origin ranged from 52.27 to 84.85 µg/g. In bee bread of different botanical origin collected from five locations in Turkey, Bayram et al. [33] reported caffeic acid concentrations ranging from 11.49 to 102.09 µg/100 g. Other authors found much lower levels in samples from the same country, ranging from 2.347 to 13.691 µg/g, while in several samples, the acid was not detected at all [13]. In the study by Kolayli et al. [23], caffeic acid was detected in only one of the eleven bee bread samples analyzed, at a level of 25.38 µg/g. In Romania, Dranca et al. [26] reported a caffeic acid concentration of 0.10 mg/L. Elsayed et al. [27] also reported low amounts of caffeic acid (4.198 µg/g) for bee bread collected in Egypt during the citrus bloom. Sawicki et al. [28] did not detect caffeic acid in Polish bee bread, and earlier research by Isidorov et al. [29], covering samples from various countries, including Poland, showed only trace amounts of this substance.

The average vanillic acid content in bee bread determined in our study was comparable between collection seasons and ranged from 51.85 µg/g to 67.84 µg/g. Our results for vanillic acid were higher than those reported by other authors. Bee bread from apiaries across seven districts of Romania contained nearly two times lower concentrations of this acid (29.27–39.39 µg/g) [14]. Bee bread of citrus origin showed a vanillic acid level of 20.32 µg/g [27]—approximately three times lower than the values obtained in our study. Aksoy et al. [12] reported very low concentrations of vanillic acid in bee bread from different regions of Turkey (0.13–0.45 µg/g), and Dranca et al. [26] did not detect this compound in Romanian bee bread.

Salicylic acid was detected in samples from the 2016 and 2017 beekeeping seasons at mean levels of 115.69 µg/g and 72.72 µg/g, respectively. In contrast, its concentration in all but two samples collected in 2015 was below the limit of detection (<0.02 µg/g). Only a few authors have reported the presence of salicylic acid in bee bread. Bayram et al. [33] found much lower salicylic acid concentrations (20.86–35.95 µg/g) in bee bread of different botanical origins collected in Turkey. Erol [39] found significant disparities in salicylic acid content between bee bread samples from two subsequent years of the study: 7.12 µg/g in 2021 and 19.44 µg/g in 2022. Kutlu et al. [40], using choline chloride-acetic acid deep eutectic solvent, obtained a value of 1.112 µg/g of the acid. We hypothesize that the presence of salicylic acid in bee bread may be associated with the occurrence of *Salix* spp. (willow) pollen, as suggested by Urcan et al. [41] for bee pollen. In Poland, *Salix* spp. constitute an important source of nectar and pollen in early spring; however, their availability to honey bees is strongly influenced by environmental conditions. This variability may partly explain the pronounced differences in salicylic acid content observed between bee bread collection years. As phenological data, palynological analysis, and weather records were not collected in the present study, further interpretation of this relationship remains beyond the scope of our work.

The average vanillin aldehyde content ranged from 22.28 µg/g in samples collected in 2015 to 143.81 µg/g in those from 2016. Extremely high variability was observed across all sampling seasons (CV = 159.8%). To the best of our knowledge, vanillin aldehyde has not been previously reported in bee bread.

Among the detected flavonoids, rutin was predominant in bee bread samples collected for our study. The average rutin content in samples from 2015 (1390.68 µg/g) was 3 to 4 times higher than that in samples from the following years. Our findings were comparable to those of Elsayed et al. [27], who reported a rutin concentration of 535.00 µg/g in citrus bee bread. Relatively high values were also observed in Romanian samples, where rutin ranged from 56.51 to 383.49 µg/g [14]. The rutin contents reported by other authors were generally much lower compared with our results. Bayram et al. [33] recorded rutin content in the range 12.26–38.04 µg/g in bee bread from five Turkish locations, attributing the variability to differences in floral sources available to foraging bees. Kolayli et al. [23] found rutin levels between 1.39 and 83.62 µg/g in bee bread from various regions of Turkey, while Aksoy et al. [12] reported very low concentrations (0.38–0.60 µg/g) or absence of rutin in samples from another Turkish region. In Polish bee bread from the Kujawy region collected in 2019, Sawicki et al. [28] detected only 5.1 µg/g of rutin. In a subsequent study, rutin content in bee bread from the same region collected in 2020 was determined at 6.96 µg/g in the free form and 3.84 µg/g in the form of glycoside bonds [42].

The second-most abundant flavonoid in bee bread samples across the studied beekeeping seasons was hesperidin. The highest content was observed in samples from 2016 (mean = 636.85 µg/g), while samples from 2015 and 2017 contained approximately one-third less. Hesperidin has been detected in all bee bread samples from various locations in Turkey analyzed by Aksoy et al. [12], though in much smaller amounts, ranging from 0.04 to 0.60 µg/g. In contrast, Ilie et al. [14] detected this flavonoid in only one out of twelve samples from Romania, at a notable concentration of 52.17 µg/g. A similar concentration was obtained by Sonmez et al. [43] for bee bread from Anatolia, Turkey.

The mean hesperetin concentration in our samples collected in 2017 (503.87 µg/g) was approximately 4 times higher than in samples from 2015 and 2016. Can et al. [13] detected hesperetin in only one out of fifteen bee bread samples from Turkey, at a concentration of 22.729 µg/g. Hesperetin was not detected in any bee bread samples analyzed by Aylanc et al. [44] from northeastern Portugal, nor was it quantified in samples from Artvin province in Turkey by Izol et al. [45].

In this study, average kaempferol levels ranged from 56.09 to 179.89 µg/g, and quercetin from 66.21 to 94.85 µg/g. In citrus bee bread from Egypt, Elsayed et al. [27] reported quercetin (717.40 µg/g) as the major flavonoid and kaempferol (34.02 µg/g) as a minor component. Kaempferol and quercetin derivatives were also identified as the dominant flavonol structures in bee bread analyzed by Bovi et al. [32], with quantities varying by season (spring, summer, autumn, winter), although the principal structures remained consistent. The quercetin concentrations determined in our study fall within the range reported by Can et al. [13] (5.701–367.720 µg/g), although these authors did not detect quercetin in all samples. Conversely, our kaempferol values align with those reported by Isidorov et al. [29] (80–400 µg/g). Bee bread samples from different regions of Romania contained much higher concentrations of these flavonoids (quercetin: 993.36–1192.04 µg/g; kaempferol: 762.24–1892.27 µg/g), as reported by Ilie et al. [14]. Similar trends were noted by Urcan et al. [31] in Romanian and Indian samples, in which kaempferol-3-O-glycosides and hydroxycinnamic acid derivatives were predominant, occurring at very high concentrations. In contrast, Bayram et al. [33] reported much lower levels of quercetin (9.71–39.18 µg/g) and kaempferol (3.80–26.81 µg/g) in Turkish bee bread than those detected by us. Very low quercetin contents (0.13–0.36 µg/g) were also reported by Aksoy et al. [12]. Dranca et al. [26] identified kaempferol as the main flavonoid in bee bread extracts (31.25 mg/L), whereas quercetin occurred at very low levels (0.06 mg/L). In bee bread from Poland, only trace amounts of kaempferol were detected by Markiewicz-Żukowska et al. [25], and Sawicki et al. [28] did not detect this compound at all. Nevertheless, our findings clearly confirm the presence of both kaempferol and quercetin in bee bread produced under Polish climatic conditions.

The highest content of isorhamnetin in our study was found in samples collected in 2016 (mean = 139.83 µg/g), while the lowest occurred in samples from 2015 (mean = 22.33 µg/g). Bayram et al. [33] found lower concentrations of isorhamnetin in Turkish samples, ranging from 3.80 to 26.81 µg/g. Sawicki et al. [28] reported very low amounts of isorhamnetin-3-O-rutinoside in Polish bee bread (2.5 µg/g), values at least ten times lower than those obtained by us. In contrast, Isidorov et al. [29] reported extremely high levels of isorhamnetin in bee bread from Poland, Russia, and Ukraine (4000–9000 µg/g). Likewise, bee bread from Romania and India contained very high amounts of isorhamnetin-3-O-glycoside (910 µg/g) according to Urcan et al. [31]. Ilie et al. [14] also detected elevated concentrations of this flavonoid in Romanian samples (464.43–1333.09 µg/g). Additionally, Sobral et al. [30] identified isorhamnetin glycoside derivatives in bee bread samples from northeastern Portugal.

The mean sum of phenolic compounds in bee bread samples collected over the three consecutive beekeeping seasons in our study ranged from 1885.50 to 2625.89 µg/g. This included total phenolic acids ranging from 444.24 to 757.16 µg/g and total flavonoids ranging from 1350.32 to 2101.02 µg/g. The study demonstrated a statistically higher total phenolic compounds concentration in samples collected during the first two years (2015 and 2016) compared to those obtained in 2017. Regarding phenolic acids, bee bread samples from 2016 contained significantly higher levels compared with samples from 2015 and 2017. The highest sum of flavonoids was recorded in samples from 2015 and 2016. Several authors have demonstrated that the qualitative and quantitative profiles of phenolic compounds in bee bread vary throughout the year, depending on the season of collection (spring, summer, autumn, winter) [32]. Chemical analysis of the flavonoid fraction conducted by these authors showed that particular groups of flavonoids predominated in specific seasons, and some were present exclusively at certain times of the year. The findings of our study are consistent with these observations: the concentrations of individual phenolic compounds in bee bread differed between the examined beekeeping seasons. Among all analyzed compounds, only *p*-coumaric and vanillic acids exhibited stability, showing no statistically significant differences between the years of collection. The observed variability in phenolic composition can be attributed to differences in the botanical diversity of pollen sources available to bees during the three sampling years. Similar conclusions were reached by other researchers investigating bee bread of various botanical and geographical origins [32,46]. However, the lack of palynological analysis of bee bread in our study, as well as in many of the studies cited above, hinders a thorough discussion of differences in the phenolic composition of bee bread and attribution of particular phenolic compounds to pollen sources from which bee bread originated.

### 2.2. Total Phenolic Content (TPC) and Antioxidant Activity

The Folin–Ciocalteu method was used to determine the total phenolic content (TPC) of bee bread samples collected over three consecutive beekeeping seasons. The TPC differed significantly depending on the season of collection (Table 2). The highest TPC value was obtained for samples collected during the 2017 season (1469.15 mg GAE/100 g), whereas the lowest was recorded for samples from 2015 (873.33 mg GAE/100 g).

In recent years, numerous studies have examined the TPC and antioxidant activity of bee bread. Reported TPC values for samples from various geographical regions range widely, from 250 to 3715 mg GAE/100 g [13,14,22,27,28,31,47,48]. These studies consistently indicate that the phenolic composition of bee bread varies substantially with geographic origin, likely reflecting differences in the botanical sources of the pollen used by bees to produce bee bread. The TPC values obtained in our study are generally comparable with those reported by other authors. For instance, TPC in bee bread from Turkey reached 2445 mg GAE/100 g as described by Keyvan et al. [22], while Can et al. [13] reported values ranging from 439.3 to 1491.7 mg GAE/100 g for samples from the same country. Bee bread from Egypt exhibited a TPC of 1071 mg GAE/100 g [27]. Ilie et al. [14] reported TPC values between 710 and 1830 mg GAE/100 g for samples from Romania, similar to the range found by Urcan et al. [31] for samples originating from Romania and India (567–1283 mg GAE/100 g). Comparable TPC values were also obtained for Polish bee bread (823 mg GAE/100 g) by Sawicki et al. [28]. In contrast, markedly higher TPC values—ranging from 3343 to 3652 mg GAE/100 g—were reported by Markiewicz-Żukowska et al. [25] for bee bread samples from Poland.

The antioxidant activity of the bee bread samples was evaluated using the DPPH assay for a fixed extract concentration (1 g/100 mL, in methanol). This approach was chosen due to the limited amount of bee bread material available and the large number of individual samples analyzed separately for each sampling year. Our aim was to enable a direct comparison of antioxidant potential among samples and years under identical experimental conditions. Samples collected over the three consecutive beekeeping seasons exhibited high antioxidant activity, with mean values of over 94% (Table 2). No statistically significant differences in antioxidant activity were observed between the sampling years. Moreover, the results were characterized by exceptionally low variability within each season, with CV values not exceeding 2.00%.

Direct comparison of our DPPH results with previously published data is challenging due to differences in reporting units and methodological approaches. Many authors express antioxidant capacity as SC_50_ (mg/mL)—the extract concentration required to scavenge 50% of DPPH radicals [13]. In contrast, our study assessed antioxidant activity based on DPPH radical scavenging efficiency expressed as a percentage. Additionally, variations in extraction solvents (e.g., ethanol, hexane, water) used to isolate antioxidant compounds further complicate direct comparison between studies. For ethanolic extracts of bee bread, Dervisoglu et al. [49] reported DPPH scavenging activity ranging from 20.15% to 93.18%, depending on extract concentration. Akhir et al. [50] obtained similar values for 70% ethanolic extracts, with an average scavenging activity of 93.60%, which aligns closely with our findings. Comparable results (mean 85.79%) were also reported by Suleiman et al. [51] for ethanolic extract. However, these authors reported ten times lower antioxidant activity in bee bread water extracts. Similarly, Keyvan et al. [22] demonstrated very low DPPH radical scavenging activity for water-soluble extracts of Turkish bee bread, with mean values of only 3.40 ± 2.99%.

For the combined dataset from 2015 to 2017, significant correlations with antioxidant activity were observed for *p*-coumaric acid (moderate positive), hesperidin and the sum of phenolic acids (weak positive), as well as hesperetin (weak negative) (Table 3). When the data were analyzed in subsets, additional variables were found to influence DPPH radical scavenging activity, including *trans*-ferulic acid, hesperidin, vanillin, quercetin, the sum of flavonoids, and the sum of phenolic compounds. In all bee bread samples analyzed in this study, the antioxidant activity exceeded 92%, regardless of the levels of TPC. This indicates that compounds other than phenolics may substantially contribute to the high antioxidant potential of bee bread. Although phenolic compounds are known to play an important role, additional constituents—such as proteins, enzymes, ascorbic acid, and carotenoids—may also enhance free-radical-scavenging activity [52].

Unlike in this study, Stanciu et al. [53] found a positive correlation between TPC and DPPH radical scavenging activity. Very strong correlation coefficient between TPC and antioxidant activity in bee bread was calculated by Ivanišová et al. [54] and Kalaycıoğlu et al. [55] as well as Aylanc et al. [44].

### 2.3. Principal Component Analysis

Five principal components with eigenvalues > 1 (according to the Kaiser criterion) accounted for 76.13% of the total variance (Supplementary Table S2). The first principal component (PC1) explained 25.96% of the variance and showed negative correlations with most variables, except for rutin and hesperetin. The second principal component (PC2), which accounted for 19.49% of the variance, was strongly positively correlated with rutin but negatively correlated with hesperetin and total phenolic content (TPC) (Table 4).

**Figure 1:**
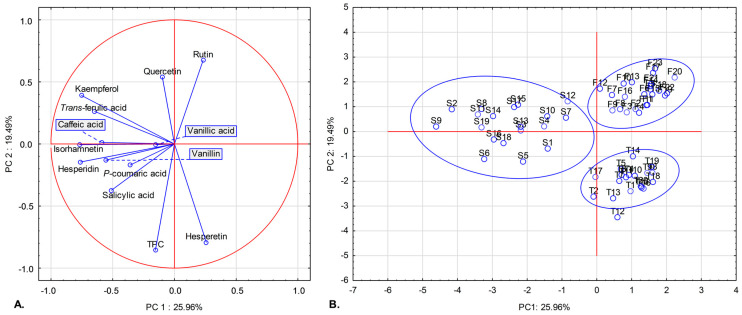
Projection of variables (**A**) and cases (**B**) in two-factor plane (PC1 × PC2). TPC—Total Phenolic Content; F1–F23—bee bread samples from 2015; S1–S19—samples collected in 2016; T1–T20—bee bread samples from 2017.

The projection of the variables onto the PC1 × PC2 plane is presented in Figure 1A. Figure 1B illustrates that almost all bee bread samples clustered into three groups, consistent with the year of collection. One sample from 2017 did not associate with any cluster. Samples collected in 2015 (F1–F23) exhibited positive scores on both PC1 and PC2 and were characterized by the highest rutin concentrations, the lowest isorhamnetin and total phenolic content (TPC) concentrations, and the absence of detectable salicylic acid (with the exception of samples F18 and F20). Bee bread samples from 2016 (S1–S19), which showed negative PC1 scores, were distinguished by increased levels of caffeic, *trans*-ferulic, and salicylic acids and by higher concentrations of hesperidin, kaempferol, and isorhamnetin. Finally, samples collected in 2017 (T1–T20), scored mostly positive with PC1 and negative with PC2, indicating the highest hesperetin and TPC concentrations.

Principal Component Analysis (PCA) reduces complex, multivariate data into principal components that are easier to interpret, allowing for visual clustering and differentiation among studied samples [56]. In this study, thirteen variables representing individual phenolic compounds and total phenolic content (TPC) were included in the PCA. The variance explained by the first two principal components (PC1 and PC2) was moderate (45.5%), but comparable to the results reported by Can et al. [13], who applied chemometric methods (PCA and hierarchical clustering) based on phenolic composition and antioxidant activity. In contrast, Kalaycıoğlu et al. [55], who incorporated aliphatic organic acids, sugar content, and DPPH radical scavenging activity into their PCA, achieved a clearer differentiation of bee bread samples from different regions of Anatolia (Turkey), with PC1 and PC2 explaining nearly 84% of the total variance. Aksoy et al. [12] included an even broader range of variables—free amino acids, phenolic compounds, organic acids, sugars, and antioxidant capacity—and reported approximately 68% of the variance explained by the first two principal components when differentiating bee bread samples from six districts of Ardahan Province, Turkey. Similarly, Çobanoğlu et al. [57] obtained a high cumulative variance (73%) for PC1 and PC2 by incorporating phenolic compounds, antioxidant capacity, and carotenoid content into their PCA. It is worth noting that, compared to the present study, the referenced studies were based on smaller sample sizes, ranging from five to thirty-seven samples. Consequently, the relatively moderate variance explained by PC1 and PC2 in our 62 samples suggests that the phenolic composition of bee bread is influenced by multiple contributing factors rather than a single dominant component.

## 3. Materials and Methods

### 3.1. Sample Collection

Bee bread samples were collected over three consecutive beekeeping seasons (2015–2017) in the experimental apiary of the National Institute of Horticultural Research, Apiculture Division, in Puławy, Poland. The field experiment methodology was described in detail by Semkiw and Skubida [35]. To enhance bee bread production, honeybee colonies were transported each year to several locations with diverse forage availability. The primary pollen sources during the study seasons included *Salix* spp., *Taraxacum officinale*, *Ribes nigrum*, *Prunus domestica*, *Malus domestica*, *Brassica napus* var. *oleifera*, and *Rubus idaeus*. Before winter, colonies were returned to a common site where they remained until spring. This location provided *Solidago* spp. and *Sinapis alba* nectar flows in autumn, and mainly *Salix* spp. and *Acer* spp. nectar in spring. Bee bread was extracted from frozen combs, purified, and stored at −20 °C until analysis. In total, 62 bee bread samples were obtained over the three study seasons. Phenolic compound composition, TPC and antioxidant activity measurements were performed within three months after collection. All quantitative data were expressed on a fresh weight basis.

### 3.2. Reagents

DPPH (2,2-diphenyl-1-picrylhydrazyl, purity ≥ 90.0%); formic acid (≥95.0%), vanillin (≥99.0%), as well as reference standards of phenolic compounds, including phenolic acids ((≥97.5%), caffeic (≥98.0%), vanillic (≥97.0%), salicylic (≥99.0%), *p*-coumaric (≥98.0%), and *trans*-ferulic acid (≥99.0%)) and flavonoids (hesperidin (≥80.0%), quercetin (≥95.0%), kaempferol (≥97.0%), isorhamnetin (≥95.0%), hesperetin (≥95.0%), rutin (≥90.0%)) were purchased from Sigma-Aldrich (Poznań, Poland). Potassium hexacyanoferrate (II), K_4_Fe(CN)_6_ × 3H_2_O, (ppa grade); and zinc acetate, Zn(CH_3_COO)_2_ × 2H_2_O, (ppa grade) used for the preparation of Carrez reagents I and II, ethyl alcohol, Folin–Ciocâlteu reagent, and anhydrous sodium carbonate (ppa grade) were purchased from POCH (Gliwice, Poland). Methanol for HPLC analyses (≥98.0%) and BakerBond C18-SPE 500 mg/6 mL solid-phase extraction columns were supplied by J.T. Baker (Dventer, The Netherlands). Ultrapure water, for both solvent and diluent, from the Milli-Q system (Barnstead, Dubuque, IA, USA; resistivity: 18.3 MΩ cm) was used.

### 3.3. Extract Preparation

A homogenized bee bread sample (5.000 ± 0.001 g) was dissolved in acidified water (deionized water containing 1% formic acid), mixed for 30 min using a mechanical shaker at room temperature, and transferred to a 50 mL volumetric flask. Following the addition of 0.5 mL each of Carrez I and Carrez II solutions, the flask was filled to volume with acidified water. The resulting suspension was then filtered through soft filter paper. Next, 10 mL of the filtrate was applied to a C18-SPE column (500 mg/6 mL, Bakerbond, VWR International, Radnor, PA, USA) preconditioned with 6 mL of methanol followed by 6 mL of acidified water. After washing the column with 6 mL of acidified water, phenolic compounds were eluted with 6 mL of methanol and collected in a 10 mL volumetric flask, which was then filled to volume with methanol. Finally, the solution was filtered through a 0.45 μm PTFE membrane filter (Carl Roth GmbH + Co. KG, Karlsruhe, Germany) and injected into the HPLC-DAD system for chromatographic analysis.

### 3.4. HPLC-DAD Analysis

Qualitative and quantitative determination of individual phenolic compounds in bee bread samples was performed using high-performance liquid chromatography with a photodiode array detector (HPLC-DAD, Shimadzu, Tokyo, Japan), following the method described by Waś et al. [58] for pollen loads, with slight modifications. The analyses were carried out on a Shimadzu HPLC-DAD system (Shimadzu, Kyoto, Japan) equipped with a photodiode array detector (SPD-M20A).

Reverse-phase chromatographic separation was conducted using a C18 column (Synergi 4 μm Fusion-RP 80A, 250 × 4.6 mm, Phenomenex Inc., Torrance, CA, USA). The separation was performed under gradient conditions with two solvent systems: A—acidified water containing 1% formic acid, and B—methanol. The gradient program was as follows: 84% A/16% B at the initial stage, 10% A/90% B at 50 min, and 84% A/16% B at 51–62 min. The flow rate was 1 mL/min, the column temperature was maintained at 40 °C, and the injection volume was 10 μL.

Individual phenolic compounds were identified based on retention times and spectral analysis in the range of 190–400 nm (Supplementary Figure S1). The following wavelengths were used for quantitative determination: 270 nm (vanillic acid, vanillin, hesperidin, rutin), 300 nm (*p*-coumaric acid, salicylic acid, hesperetin), and 320 nm (caffeic acid, *trans*-ferulic acid, quercetin, kaempferol, isorhamnetin). Quantitative analysis was performed using the external standard method (Supplementary Figures S2 and S3). Each sample was analyzed in triplicate, and the mean value was calculated.

### 3.5. Determination of Total Phenolic Content (TPC)

The total phenolic content (TPC) of bee bread extracts was determined using the Folin–Ciocâlteu method, following Meda et al. [59] with slight modifications. The method is based on the ability of phenolic compounds to form a blue complex with the Folin–Ciocâlteu reagent and a saturated sodium carbonate (Na_2_CO_3_) solution. Briefly, 1 g of bee bread was dissolved in a small volume of 70% ethanol, followed by the addition of 0.5 mL each of Carrez I and Carrez II reagents, and the solution was brought to 50 mL. The mixture was shaken mechanically for 30 min and then filtered through soft filter paper. Subsequently, 0.5 mL of the extract was mixed with 2.5 mL of 10% Folin–Ciocâlteu reagent. After 5 min, 2 mL of 0.7 M Na_2_CO_3_ was added, and the mixture was incubated in the dark at room temperature for 2 h. Absorbance was measured at 760 nm against a blank using a spectrophotometer (SPEKORD 200, Analytik Jena, Jena, Germany). Quantification was performed using a gallic acid calibration curve (0.025–0.15 mg/mL, R^2^ = 0.9992). Results were expressed as mg gallic acid equivalents (GAE) per 100 g of extract. Each measurement was conducted in triplicate, and the mean value was reported.

### 3.6. Determination of Antioxidant Activity Against the DPPH Radical

The radical scavenging activity of bee bread extracts was evaluated using the 2,2-diphenyl-1-picrylhydrazyl (DPPH) assay, following Meda et al. [59] with minor modifications. Briefly, 0.5 g of homogenized bee bread was extracted with 50 mL of methanol on a mechanical shaker for 30 min and filtered through medium-porosity filter paper. Then, 0.75 mL of the extract was mixed with 1.5 mL of DPPH solution in methanol (0.02 mg/mL) and incubated in the dark at room temperature for 15 min. Absorbance was measured at 517 nm using a spectrophotometer (Spekord 200, Analytic Jena). A methanol blank was included, and each sample was analyzed in triplicate. Radical scavenging activity was calculated as the percentage of DPPH discoloration following the equation

$$DPPH\;\left[\%\right]=\left(\frac{A_{0}-A_{x}}{A_{0}}\right)\times 100$$

where A_0_—the absorbance of the control; A_x_—the absorbance for bee bread solution.

### 3.7. Statistical Analysis

In our dataset, measurements for seven phenolic compounds (vanillic acid, salicylic acid, vanillin, rutin, hesperetin, kaempferol, and isorhamnetin) included values below the limit of detection (LOD = 0.02 µg/g for all compounds) (Supplementary Table S1). To avoid excluding these variables from multivariate analysis or assigning zero values, non-detectable concentrations were replaced with a low imputed value (LOD/2^0.5^). This conservative substitution was applied uniformly across compounds to minimize artificial inflation of variance while preserving relative differences among samples in subsequent chemometric analyses, as suggested in [60] and applied in other chemometric studies, e.g., [61,62]. The assumptions of distribution normality were not met (according to Shapiro–Wilk test) and the data were compared between bee bread collection seasons using Kruskal–Wallis test. The correlations between individual phenolic compounds, TPC, and antioxidant activity were calculated and expressed as Spearman’s rank correlation coefficients. Variability in phenolic compound composition between bee bread samples was illustrated using Principal Component Analysis (PCA). The dataset was first processed by excluding aggregate variables (sum of phenolic acids, sum of flavonoids, sum of phenolic compounds) to avoid redundancy, as well as removing antioxidant capacity—the variable with extremely low variance (CV = 1.32%, Table 2). Due to the fact that variables were expressed in different units, prior to the analysis, the variables were autoscaled (mean-centered and scaled to unit variance). PCA was then performed on the autoscaled dataset. The number of principal components retained for interpretation was determined according to the Kaiser criterion, whereby components with eigenvalues greater than 1 were considered significant [56]. For visualization purposes, PCA score plots were constructed using the first two principal components. K-means clustering (k = 3), selected based on the elbow method and supported by the clustering structure observed in the PCA score plots, was applied to the PCA scores to identify sample groupings, with 95% confidence ellipses added to the plots. The statistical analyses were performed using Statistica ver. 13.3 (TIBCO Software Inc., Palo Alto, CA, USA).

## 4. Conclusions

Vanillin aldehyde, caffeic, *p*-coumaric, *trans*-ferulic, salicylic, and vanillic acids, as well as the flavonoids rutin, kaempferol, hesperidin, quercetin, isorhamnetin, and hesperetin, were identified and quantified in most bee bread samples. Among the phenolic acids, the highest concentrations were observed for *p*-coumaric acid, followed by *trans*-ferulic and caffeic acids. In the group of the flavonoids, rutin predominated, followed by hesperidin. Seasonal variation was observed in the levels of individual phenolic compounds in bee bread, except for *p*-coumaric and vanillic acids.

The total phenolic content (TPC) averaged 1166.05 mg GAE/100 g and differed significantly between the seasons of bee bread collection. Our study demonstrated that bee bread collected over the three beekeeping seasons exhibited high antioxidant activity (DPPH radical scavenging activity; mean = 94.7%). No significant differences in antioxidant activity were observed among the corresponding years of sampling. Moderately strong positive correlation between *p*-coumaric acid content and antioxidant activity was found across years 2015–2017. A weak influence of the contents of hesperetin, hesperidin, and the sum of phenolic compounds quantified on antioxidant activity was also revealed. The absence of a significant influence of TPC on DPPH radical scavenging activity indicates that non-phenolic compounds may play an important role in the observed antioxidant capacity.

The Principal Component Analysis indicated that all samples except one were grouped into three distinct clusters based on their year of collection. The variability observed in TPC and in the concentrations of individual phenolic compounds is likely to be attributed to differences in the botanical origin of pollen available to the honey bees over the three-year sampling period.

Our findings on the phenolic composition and antioxidant capacity of bee bread expand current knowledge of the chemical and nutritional profile of this product under the climatic conditions of Poland. The results strengthen the scientific premise for future studies evaluating the potential application of bee bread as a functional food.

## Figures and Tables

**Table 1:** Content (µg/g) of phenolic compounds in bee bread samples. Mean ± standard deviation along with coefficient of variation expressed as a percentage (in brackets) is given.

Phenolic Compound	2015 (*n* = 23)	2016 (*n* = 19)	2017 (*n* = 20)	2015–2017 (*n* = 62)	Test Statistic H	*p* Value
*p*-Coumaric Acid	195.02 ± 93.42 ^A^ (47.90%)	274.63 ± 235.64 ^A^ (85.80%)	210.58 ± 76.61 ^A^ (36.38%)	224.44 ± 150.11 (66.88%)	0.336	0.846
*trans*-Ferulic Acid	87.53 ± 26.70 ^B^ (30.50%)	187.15 ± 100.43 ^C^ (53.66%)	40.73 ± 8.52 ^A^ (20.91%)	102.96 ± 82.60 (80.22%)	51.363	<0.001
Caffeic Acid	69.80 ± 25.07 ^A^ (35.92%)	111.85 ± 33.69 ^B^ (30.12%)	72.51 ± 39.89 ^A^ (55.01%)	83.56 ± 37.66 (45.07%)	20.020	<0.001
Vanillic Acid	58.22 ± 32.27 ^A^ (55.43%)	67.84 ± 27.83 ^A^ (41.02%)	51.85 ± 68.78 ^A^ (132.67%)	59.11 ± 46.03 (77.88%)	4.408	0.110
Salicylic Acid	<LOD *	115.69 ± 67.21 ^B^ (58.09%)	72.72 ± 86.93 ^A^ (119.54%)	60.44 ± 77.20 (127.73%)	32.909	<0.001
Vanillin	22.28 ± 17.76 ^A^ (79.69%)	143.81 ± 171.09 ^AB^ (118.97%)	46.19 ± 14.09 ^B^ (30.51%)	67.23 ± 107.45 (159.82%)	8.630	0.013
Rutin	1390.68 ± 773.55 ^C^ (55.62%)	563.35 ± 281.61 ^B^ (49.99%)	270.61 ± 181.41 ^A^ (67.04%)	775.83 ± 699.82 (90.20%)	33.607	<0.001
Hesperidin	323.97 ± 175.93 ^A^ (54.31%)	636.85 ± 96.52 ^B^ (15.16%)	410.43 ± 86.59 ^A^ (21.10%)	447.74 ± 183.35 (40.95%)	33.527	<0.001
Hesperetin	128.06 ± 86.32 ^A^ (67.41%)	141.72 ± 164.93 ^A^ (116.38%)	503.87 ± 121.22 ^B^ (24.06%)	253.48 ± 213.70 (84.31%)	33.934	<0.001
Kaempferol	96.95 ± 39.82 ^B^ (41.07%)	179.89 ± 76.64 ^C^ (42.60%)	56.09 ± 17.82 ^A^ (31.77%)	109.19 ± 70.30 (64.38%)	33.714	<0.001
Quercetin	89.80 ± 26.72 ^B^ (29.76%)	94.85 ± 36.89 ^B^ (38.89%)	66.21 ± 17.64 ^A^ (26.65%)	83.74 ± 30.15 (36.01%)	9.826	0.007
Isorhamnetin	22.33 ± 8.48 ^A^ (37.99%)	139.83 ± 88.20 ^C^ (63.08%)	44.62 ± 12.36 ^B^ (27.71%)	65.53 ± 70.26 (107.22%)	42.987	<0.001
Sum of phenolic acids	414.68 ± 80.04 ^A^ (19.30%)	757.16 ± 269.34 ^B^ (35.57%)	448.40 ± 131.37 ^A^ (29.30%)	530.51 ± 228.83 (43.13%)	29.793	<0.001
Sum of flavonoids	2051.79 ± 861.80 ^B^ (42.00%)	1756.49 ± 369.07 ^B^ (21.01%)	1351.82 ± 275.39 ^A^ (20.37%)	1735.50 ± 646.37 (37.24%)	10.191	0.006
Sum of phenolic compounds	2488.75 ± 885.68 ^B^ (35.59%)	2657.47 ± 539.13 ^B^ (20.29%)	1846.40 ± 315.41 ^A^ (17.08%)	2333.24 ± 720.58 (30.88%)	15.803	<0.001

* Salicylic acid was quantified only in 2 samples in 2015. LOD—limit of detection. Values that differ significantly according to the Kruskal–Wallis test are indicated by different letters and should be compared within each table row.

**Table 2:** Total phenolic content and antioxidant activity (expressed as DPPH radical scavenging activity) of bee bread samples. Mean ± standard deviation along with coefficient of variation (in brackets) is given.

Characteristics	2015 (*n* = 23)	2016 (*n* = 19)	2017 (*n* = 20)	2015–2017 (*n* = 62)	Test Statistic H	*p* Value
Total phenolic content (TPC) (mg GAE/100 g)	873.33 ± 113.52 ^A^ (13.00%)	1201.35 ± 85.65 ^B^ (7.13%)	1469.15 ± 110.87 ^C^ (7.55%)	1166.05 ± 271.03 (23.24%)	51.708	<0.001
DPPH radical scavenging activity (%)	94.40 ± 1.50 ^A^ (1.59%)	95.04 ± 0.88 ^A^ (0.93%)	94.69 ± 1.21 ^A^ (1.28%)	94.69 ± 1.25 (1.32%)	1.263	0.532

Values that differ significantly according to the Kruskal–Wallis test are indicated by different letters and should be compared within each table row.

**Table 3:** Correlation coefficients (Spearman’s rho) between particular phenolic compounds, sum of phenolic compounds and total phenolic content and antioxidant activity (expressed as DPPH radical scavenging activity).

Variable	Correlation Coefficient with DPPH Radical Scavenging Activity							
	2015		2016		2017		2015–2017	
	*rho*	*p*	*rho*	*p*	*rho*	*p*	*rho*	*p*
*p*-Coumaric Acid	0.724 *	<0.001	0.335	0.161	**0.523**	0.018	**0.579**	<0.001
*trans*-Ferulic Acid	**−0.530**	0.009	−0.280	0.245	0.305	0.190	−0.017	0.896
Caffeic Acid	−0.174	0.427	−0.350	0.142	0.122	0.609	−0.050	0.698
Vanillic Acid	0.216	0.323	−0.375	0.114	−0.431	0.058	−0.121	0.349
Salicylic Acid	-	-	0.320	0.182	−0.302	0.196	0.009	0.947
Vanillin	−0.006	0.978	**−0.463**	0.046	−0.114	0.631	−0.158	0.220
Rutin	−0.081	0.715	**−0.499**	0.030	0.193	0.415	−0.135	0.295
Hesperidin	**0.529**	0.009	0.182	0.455	0.375	0.103	**0.364**	0.004
Hesperetin	−0.344	0.108	−0.253	0.296	0.089	0.710	**−0.271**	0.033
Kaempferol	0.206	0.345	−0.121	0.621	0.006	0.980	0.099	0.446
Quercetin	**−0.575**	0.004	−0.118	0.629	−0.144	0.546	−0.214	0.095
Isorhamnetin	0.103	0.639	0.038	0.877	0.237	0.314	0.133	0.304
Sum of phenolic acids	**0.695**	<0.001	−0.080	0.743	−0.054	0.821	**0.290**	0.022
Sum of flavonoids	0.068	0.759	**−0.604**	0.006	0.237	0.315	−0.001	0.991
Sum of phenolic compounds	0.105	0.633	**−0.561**	0.012	0.306	0.189	0.040	0.755
Total phenolic content (TPC)	0.052	0.812	−0.249	0.303	0.117	0.624	0.014	0.914

“-” not calculated because salicylic acid was quantified only in 2 samples in 2015. * Values in bold are significant at α = 0.05.

**Table 4:** Correlations between the principal components and the original variables.

Variable	Principal Component				
	1	2	3	4	5
*p*-Coumaric Acid	−0.36	−0.17	0.01	−0.77	−0.20
*trans*-Ferulic Acid	−0.65	0.26	−0.54	0.18	0.00
Caffeic Acid	−0.59	0.01	−0.56	0.06	0.26
Vanillic Acid	−0.15	−0.01	−0.06	0.44	−0.86
Salicylic Acid	−0.51	−0.37	0.29	0.26	−0.08
Vanillin	−0.56	−0.13	−0.64	−0.19	−0.14
Rutin	0.23	0.67	−0.27	−0.10	0.04
Hesperidin	−0.76	−0.15	0.32	−0.26	0.01
Hesperetin	0.26	−0.79	−0.32	0.20	0.05
Kaempferol	−0.75	0.39	0.32	0.02	0.01
Quercetin	−0.10	0.54	0.03	0.49	0.23
Isorhamnetin	−0.77	−0.01	0.34	0.22	0.15
Total phenolic content (TPC)	−0.15	−0.86	−0.01	0.22	0.23

## Data Availability

The original contributions presented in this study are included in the article and Supplementary Materials. Further inquiries can be directed to the corresponding author.

## Supplementary Materials

The following supporting information can be downloaded at: https://www.mdpi.com/article/10.3390/molecules31020304/s1, Table S1: Content (µg/g) of phenolic compounds, TPC (mg GAE/100 g) and antioxidant activity (DPPH radical scavenging activity, %) in bee bread samples collected in 2015–2017; Table S2: Eigenvalues and the proportion of variation (%) explained by the principal components; Figure S1: Diode array absorption spectra (190–40 nm) of phenolic compounds analyzed in bee bread samples; Figure S2: HPLC-DAD chromatograms of phenolic compounds: (A) of the mixture of standards, (B) of a bee bread extract; Figure S3: Calibration curves of the studied phenolic compounds in bee bread samples.
