# Supplementary material for: Phenolic Composition and Antioxidant Properties of Bee Bread Collected in Three Consecutive Beekeeping Seasons in Poland
Source: Molecules. 2026 Jan 15;31(2):304. doi: 10.3390/molecules31020304 (PMC12844223; doi:10.3390/molecules31020304)
Supplement: Supplementary file 1 [file molecules-31-00304-s001.zip › molecules-4046095-supplementary.pdf]

Table S1. Content (µg/g) of phenolic compounds, TPC (mg GAE/100 g) and antioxidant activity (DPPH radical scavenging activity, %) in bee bread samples collected in 2015-2017.

Sample name	Year of collection	*p*-Coumaric acid	*trans*-Ferulic acid	Caffeic acid	Vanillic acid	Salicylic acid	Vanillin	Rutin	Hesperidin	Hesperetin	Kaempferol	Quercetin	Isorhamnetin	Sum of phenolic acids	Sum of flavonoids	Sum of phenolic compounds	TPC	Antioxidant capacity
**F1**	**2015**	254.10	91.20	63.10	62.30	<LOD	19.40	1482.10	402.30	75.50	65.40	46.20	<LOD	470.72	2071.52	2561.64	858.71	96.12
**F2**	**2015**	334.20	80.40	71.20	69.40	<LOD	23.30	974.20	345.20	119.30	86.20	56.80	22.00	555.22	1603.70	2182.22	806.40	95.28
**F3**	**2015**	220.40	55.30	51.40	107.20	<LOD	39.40	433.20	456.20	<LOD	93.10	78.90	23.40	434.32	1084.82	1558.54	961.96	95.78
**F4**	**2015**	239.20	65.10	101.20	54.40	<LOD	16.40	1565.20	402.40	352.30	61.30	102.80	23.70	459.92	2507.70	2984.02	1012.76	95.72
**F5**	**2015**	257.00	91.20	51.30	50.40	<LOD	18.20	1804.20	348.40	145.00	87.20	68.20	23.10	449.92	2476.10	2944.22	734.24	95.86
**F6**	**2015**	234.20	53.20	31.10	50.10	<LOD	8.10	599.30	249.20	<LOD	154.30	90.40	22.80	368.62	1116.02	1492.74	875.69	94.89
**F7**	**2015**	266.40	88.40	90.40	67.10	<LOD	17.20	1401.10	567.30	72.20	101.20	80.70	20.50	512.32	2243.00	2772.52	847.40	95.50
**F8**	**2015**	289.40	83.30	96.20	58.20	<LOD	44.20	1450.40	370.30	137.70	120.40	60.10	23.60	527.12	2162.50	2733.82	1017.60	95.36
**F9**	**2015**	205.20	95.30	105.30	58.10	<LOD	15.40	911.00	505.20	179.10	106.20	78.90	21.00	463.92	1801.40	2280.72	929.70	94.92
**F10**	**2015**	236.30	74.10	56.20	56.20	<LOD	9.20	2025.10	565.40	182.00	133.40	108.20	26.90	422.82	3041.00	3473.02	885.17	92.99
**F11**	**2015**	227.30	72.40	52.40	50.10	<LOD	2.10	1703.10	434.40	140.40	93.10	46.30	23.80	402.22	2441.10	2845.42	891.47	94.52

Sample name	Year of collection	*p*-Coumaric acid	*trans*-Ferulic acid	Caffeic acid	Vanillic acid	Salicylic acid	Vanillin	Rutin	Hesperidin	Hesperetin	Kaempferol	Quercetin	Isorhamnetin	Sum of phenolic acids	Sum of flavonoids	Sum of phenolic compounds	TPC	Antioxidant capacity
**F12**	**2015**	265.30	92.40	86.20	56.30	<LOD	21.40	2138.10	560.50	117.40	187.30	50.20	36.50	500.22	3090.00	3611.62	873.74	95.02
**F13**	**2015**	212.40	102.10	70.10	60.20	<LOD	11.30	2305.40	390.30	189.30	114.30	100.90	44.00	444.82	3144.20	3600.32	942.04	95.32
**F14**	**2015**	263.10	67.30	45.30	51.40	<LOD	10.40	703.20	348.40	<LOD	58.20	107.20	20.50	427.12	1237.52	1675.04	646.15	95.75
**F15**	**2015**	243.20	65.40	70.40	68.20	<LOD	18.30	2461.30	423.40	287.50	63.20	78.30	23.80	447.22	3337.50	3803.02	844.19	94.88
**F16**	**2015**	265.20	51.20	25.20	56.30	<LOD	10.20	242.40	403.80	26.00	192.40	78.50	23.60	397.92	966.70	1374.82	733.73	95.64
**F17**	**2015**	166.10	80.00	48.30	55.10	<LOD	14.10	2507.20	280.20	32.60	101.40	120.10	28.20	349.52	3069.70	3433.32	1100.45	94.84
**F18**	**2015**	32.00	99.30	57.40	132.40	30.30	14.20	612.30	82.10	145.60	78.40	121.20	28.90	351.40	1068.50	1434.10	785.64	92.09
**F19**	**2015**	49.40	93.20	98.40	<LOD	<LOD	43.10	447.00	63.30	134.50	45.70	121.50	17.70	241.03	829.70	1113.83	828.81	92.17
**F20**	**2015**	40.10	100.40	69.40	52.40	63.80	19.40	1908.10	27.00	158.70	54.50	120.10	19.60	326.10	2288.00	2633.50	814.62	92.04
**F21**	**2015**	82.20	102.40	83.20	123.10	<LOD	8.40	762.40	93.20	127.80	100.20	123.80	16.60	390.91	1224.00	1623.31	804.10	92.04
**F22**	**2015**	34.40	166.30	56.20	<LOD	<LOD	80.40	663.20	59.30	152.30	56.20	100.02	9.89	256.93	1040.91	1378.24	787.44	92.01
**F23**	**2015**	68.40	143.40	125.40	<LOD	<LOD	48.40	2886.20	73.40	170.20	76.30	126.00	13.40	337.23	3345.50	3731.13	1104.58	92.54
**S1**	**2016**	177.00	122.70	57.00	67.30	45.00	170.00	353.00	781.90	123.40	68.10	45.00	192.00	469.00	1563.40	2202.40	1173.30	95.60

**Sample name**	**Year of collection**	***p*-Coumaric acid**	***trans*-Ferulic acid**	**Caffeic acid**	**Vanillic acid**	**Salicylic acid**	**Vanillin**	**Rutin**	**Hesperidin**	**Hesperetin**	**Kaempferol**	**Quercetin**	**Isorhamnetin**	**Sum of phenolic acids**	**Sum of flavonoids**	**Sum of phenolic compounds**	**TPC**	**Antioxidant capacity**
**S2**	**2016**	395.00	188.00	100.00	65.40	152.00	<LOD	203.10	655.10	35.40	301.40	119.10	298.40	900.40	1612.50	2512.92	1184.40	95.30
**S3**	**2016**	312.00	142.80	91.00	34.20	161.40	<LOD	523.00	556.30	67.40	193.20	82.40	195.30	741.40	1617.60	2359.02	1275.30	95.70
**S4**	**2016**	399.00	134.70	104.00	25.80	108.00	<LOD	225.20	752.30	56.20	160.40	100.00	34.00	771.50	1328.10	2099.62	1155.40	95.70
**S5**	**2016**	573.00	229.60	128.00	69.10	337.00	<LOD	413.30	611.60	125.40	56.40	51.30	29.80	1336.70	1287.80	2624.52	1107.60	95.60
**S6**	**2016**	945.00	163.50	118.00	48.60	38.00	593.00	229.10	623.30	150.20	118.10	61.20	30.20	1313.10	1212.10	3118.20	1161.70	95.30
**S7**	**2016**	208.00	158.40	92.00	55.30	109.40	<LOD	526.20	543.10	120.40	112.20	134.00	89.40	623.10	1525.30	2148.42	1240.50	95.60
**S8**	**2016**	345.00	165.60	122.00	83.40	84.20	198.20	785.30	672.50	78.20	245.30	126.30	156.20	800.20	2063.80	3062.20	1268.80	95.50
**S9**	**2016**	672.00	145.20	145.00	76.50	98.30	245.30	678.10	783.40	56.40	278.20	78.20	194.30	1137.00	2068.60	3450.90	1205.10	94.60
**S10**	**2016**	198.00	123.40	69.00	45.20	125.40	<LOD	345.40	734.60	98.40	179.10	128.40	92.40	561.00	1578.30	2139.32	1163.30	94.80
**S11**	**2016**	78.00	168.70	78.00	51.80	167.30	<LOD	746.10	692.40	82.30	267.40	94.00	326.20	543.80	2208.40	2752.22	1257.30	95.10
**S12**	**2016**	95.00	152.40	95.00	100.20	76.40	<LOD	824.20	561.80	122.10	125.40	172.20	86.40	519.00	1892.10	2411.12	1313.90	94.80
**S13**	**2016**	114.00	123.50	114.00	98.10	87.20	225.30	554.40	572.80	73.20	178.40	89.30	146.20	536.80	1614.30	2376.40	1291.60	95.20
**S14**	**2016**	154.00	142.80	154.00	50.10	104.40	189.20	369.40	611.40	59.40	243.20	67.40	156.30	605.30	1507.10	2301.60	1013.40	95.30

Sample name	Year of collection	*p*-Coumaric acid	*trans*-Ferulic acid	Caffeic acid	Vanillic acid	Salicylic acid	Vanillin	Rutin	Hesperidin	Hesperetin	Kaempferol	Quercetin	Isorhamnetin	Sum of phenolic acids	Sum of flavonoids	Sum of phenolic compounds	TPC	Antioxidant capacity
**S15**	**2016**	87.00	142.50	87.00	67.40	92.30	<LOD	426.30	742.60	65.20	305.20	78.30	125.40	476.20	1743.00	2219.22	1037.80	95.40
**S16**	**2016**	98.00	183.40	98.00	68.20	135.30	178.20	458.10	631.20	91.20	156.40	62.40	267.40	582.90	1666.70	2427.80	1299.70	95.50
**S17**	**2016**	126.00	143.60	126.00	63.10	168.20	177.30	768.30	641.20	84.30	187.20	145.00	90.30	626.90	1916.30	2720.50	1170.00	95.30
**S18**	**2016**	119.00	469.00	198.20	67.00	52.30	330.40	1241.00	437.20	582.40	86.10	42.40	78.20	905.50	2467.30	3703.20	1211.80	92.70
**S19**	**2016**	123.00	456.00	149.00	152.20	56.10	425.30	1034.20	495.40	621.20	156.20	125.30	68.40	936.30	2500.70	3862.30	1294.70	92.70
**T1**	**2017**	205.90	49.93	85.99	45.12	78.45	45.40	241.44	345.20	560.10	50.50	40.80	56.20	465.39	1294.24	1805.04	1466.23	94.58
**T2**	**2017**	250.75	43.71	76.80	78.14	300.45	34.30	452.80	456.80	594.00	51.15	90.80	54.20	749.85	1699.75	2483.90	1496.85	95.02
**T3**	**2017**	215.10	45.12	52.96	<LOD	85.68	69.10	365.60	580.20	608.40	56.47	102.40	45.60	398.88	1758.67	2226.65	1387.86	94.84
**T4**	**2017**	250.20	37.90	96.80	65.15	<LOD	78.90	202.02	350.60	445.60	49.03	78.90	42.10	450.08	1168.25	1697.23	1549.35	94.84
**T5**	**2017**	244.44	52.69	94.33	<LOD	<LOD	59.40	157.98	430.80	366.20	70.10	67.90	49.80	391.49	1142.78	1593.67	1490.82	95.12
**T6**	**2017**	244.78	43.75	63.78	110.14	76.12	34.30	358.51	500.20	506.00	62.72	50.10	49.60	538.56	1527.13	2099.99	1252.39	94.99
**T7**	**2017**	273.36	36.05	53.91	<LOD	126.45	41.40	253.60	545.60	472.75	72.57	54.20	34.50	489.78	1433.22	1964.40	1318.66	95.38
**T8**	**2017**	277.63	51.16	65.68	<LOD	<LOD	54.20	322.65	340.60	499.90	<LOD	100.70	45.90	394.51	1309.77	1758.48	1529.03	95.28

Sample name	Year of collection	*p*-Coumaric acid	*trans*-Ferulic acid	Caffeic acid	Vanillic acid	Salicylic acid	Vanillin	Rutin	Hesperidin	Hesperetin	Kaempferol	Quercetin	Isorhamnetin	Sum of phenolic acids	Sum of flavonoids	Sum of phenolic compounds	TPC	Antioxidant capacity
**T9**	**2017**	201.73	39.69	80.00	<LOD	<LOD	36.70	601.01	311.40	472.75	54.19	78.10	46.80	321.47	1564.24	1922.41	1649.81	95.47
**T10**	**2017**	260.77	46.62	52.44	49.18	78.14	50.80	180.07	325.40	329.10	44.47	68.30	45.20	487.15	992.54	1530.48	1529.32	95.45
**T11**	**2017**	279.36	52.10	82.32	<LOD	<LOD	30.20	127.64	502.60	325.40	42.22	49.80	46.80	413.82	1094.46	1538.49	1524.42	95.50
**T12**	**2017**	301.89	43.05	58.73	111.17	168.34	50.10	361.64	408.90	765.20	74.30	34.50	39.60	683.18	1684.13	2417.41	1596.28	95.48
**T13**	**2017**	265.58	36.15	54.24	120.15	225.14	34.50	162.26	423.80	542.50	51.38	60.40	36.70	701.26	1277.03	2012.79	1439.31	95.54
**T14**	**2017**	223.19	42.28	62.09	<LOD	<LOD	47.30	386.90	508.30	385.70	64.50	62.10	56.80	327.60	1464.31	1839.21	1216.53	95.58
**T15**	**2017**	231.86	41.64	63.90	<LOD	<LOD	46.80	275.22	450.50	596.60	51.42	58.80	50.20	337.43	1482.75	1866.98	1538.91	95.55
**T16**	**2017**	199.57	41.02	61.15	<LOD	<LOD	32.10	288.83	428.60	745.10	77.51	70.10	56.10	301.78	1666.24	2000.12	1521.40	95.53
**T17**	**2017**	52.53	36.06	225.60	56.23	46.18	67.20	<LOD	344.20	423.80	56.80	64.80	38.30	416.61	927.92	1411.72	1507.09	92.93
**T18**	**2017**	80.79	20.20	37.51	256.24	<LOD	40.60	<LOD	343.79	567.20	45.70	65.40	44.50	394.76	1066.61	1501.97	1451.66	92.44
**T19**	**2017**	84.24	28.16	47.18	<LOD	123.18	45.20	674.05	349.99	435.20	86.20	58.90	<LOD	282.78	1604.36	1932.34	1368.27	92.10
**T20**	**2017**	68.01	27.36	34.79	145.26	146.17	25.20	<LOD	261.03	435.90	60.50	67.10	53.40	421.58	877.95	1324.74	1548.71	92.18

Table S2. Eigenvalues and the proportion of variation (%) explained by the principal components.

Component	Eigenvalue	Proportion (%)	Cumulative (%)
1	3.37	25.96	25.96
2	2.53	19.49	45.45
3	1.60	12.33	57.78
4	1.39	10.68	68.46
5	1.01	7.67	76.13
6	0.68	5.34	81.47
7	0.66	5.04	86.51
8	0.51	3.95	90.46
9	0.41	3.19	93.65
10	0.30	2.31	95.96
11	0.22	1.65	97.61
12	0.20	1.52	99.14
13	0.11	0.86	100.00

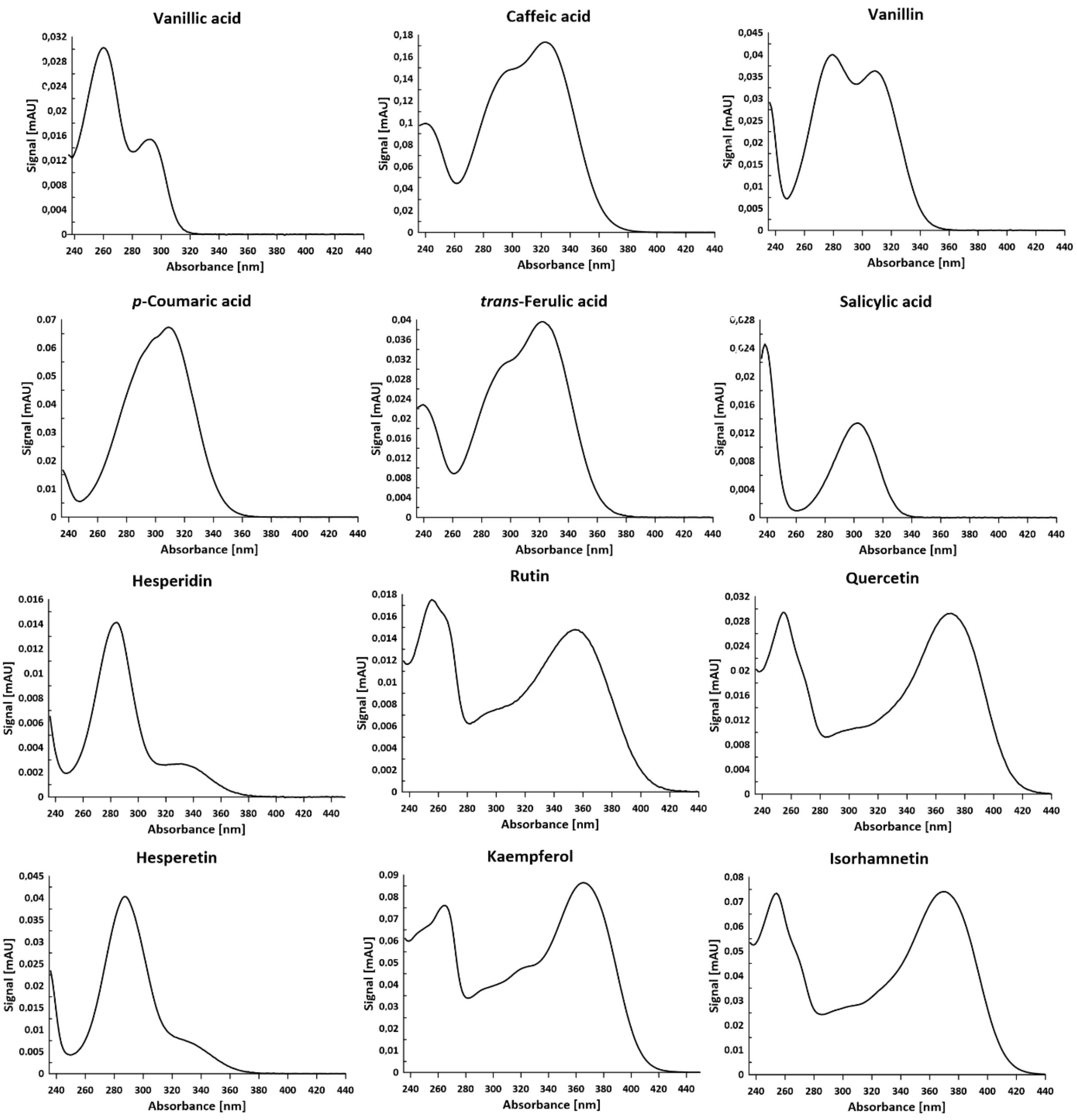

Figure S1. Diode array absorption spectra (190-40 nm) of phenolic compounds analyzed in bee bread samples.

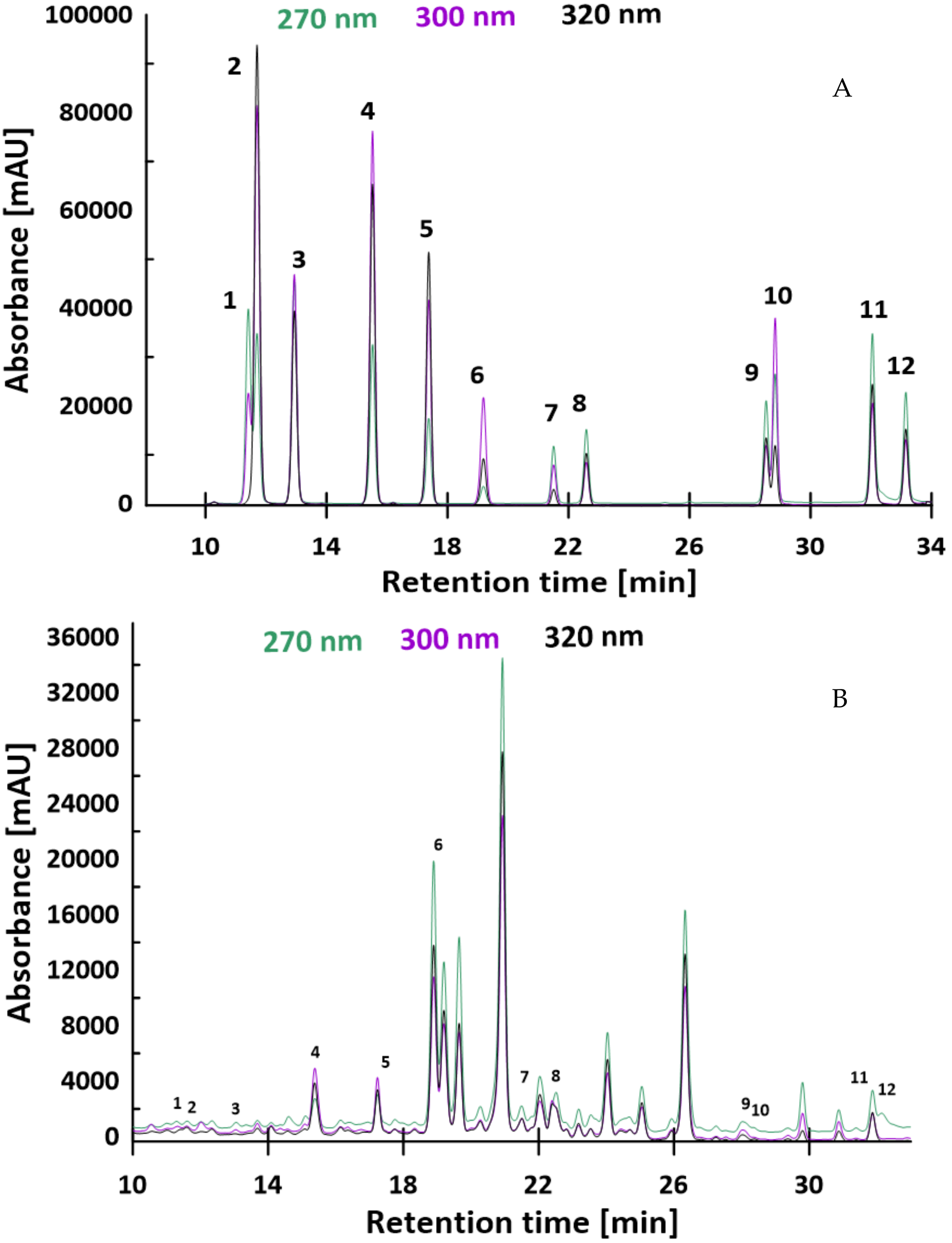

Figure S2. HPLC-DAD chromatograms of phenolic compounds: (A) of the mixture of standards, (B) of a bee bread extract. The peaks correspond to the following: (1) vanillic acid; (2) caffeic acid; (3) vanillin; (4) *p*-coumaric acid; (5) *trans*-ferulic acid; (6) salicylic acid; (7) hesperidin; (8) rutin; (9) quercetin; (10) hesperetin; (11) kaempferol; (12) isorhamnetin.

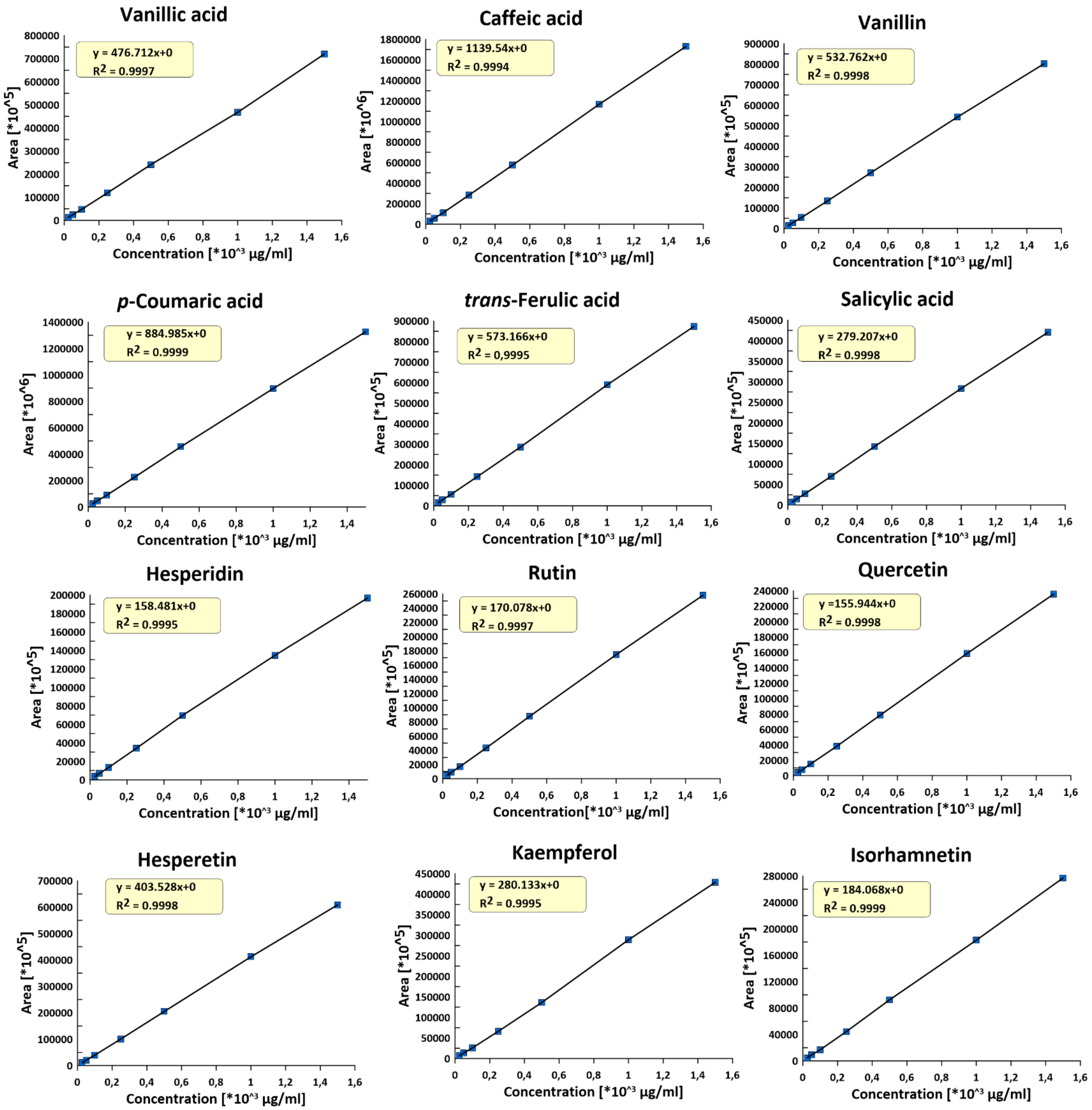

Figure S3. Calibration curves of the studied phenolic compounds in bee bread samples.
